# Acclimation of metabolism to light in *A* *rabidopsis thaliana*: the glucose 6‐phosphate/phosphate translocator GPT2 directs metabolic acclimation

**DOI:** 10.1111/pce.12495

**Published:** 2015-01-25

**Authors:** BETH C. DYSON, J. WILLIAM ALLWOOD, REGINA FEIL, YUN XU, MATTHEW MILLER, CAROLINE G. BOWSHER, ROYSTON GOODACRE, JOHN E. LUNN, GILES N. JOHNSON

**Affiliations:** ^1^ Faculty of Life SciencesUniversity of ManchesterManchesterM13 9PTUK; ^2^ School of Chemistry and Manchester Institute of BiotechnologyUniversity of ManchesterManchesterM1 7DNUK; ^3^ Max Planck Institute of Molecular Plant Physiology14476Potsdam‐GolmGermany; ^4^ Present address: Department of Animal and Plant SciencesUniversity of SheffieldAlfred Denny Building, Western BankSheffieldS10 2TNUK; ^5^ Present address: Clinical and Environmental MetabolomicsSchool of BioscienceUniversity of BirminghamEdgbastonBirminghamB15 2TTUK

**Keywords:** high light, sugar signalling, photosynthesis

## Abstract

Mature leaves of plants transferred from low to high light typically increase their photosynthetic capacity. In *A* *rabidopsis thaliana*, this dynamic acclimation requires expression of GPT2, a glucose 6‐phosphate/phosphate translocator. Here, we examine the impact of GPT2 on leaf metabolism and photosynthesis. Plants of wild type and of a GPT2 knockout (*gpt2.2)* grown under low light achieved the same photosynthetic rate despite having different metabolic and transcriptomic strategies. Immediately upon transfer to high light, *gpt2.2* plants showed a higher rate of photosynthesis than wild‐type plants (35%); however, over subsequent days, wild‐type plants acclimated photosynthetic capacity, increasing the photosynthesis rate by 100% after 7 d. Wild‐type plants accumulated more starch than *gpt2.2* plants throughout acclimation. We suggest that GPT2 activity results in the net *import* of glucose 6‐phosphate from cytosol to chloroplast, increasing starch synthesis. There was clear acclimation of metabolism, with short‐term changes typically being reversed as plants acclimated. Distinct responses to light were observed in wild‐type and *gpt2.2* leaves. Significantly higher levels of sugar phosphates were observed in *gpt2.2*. We suggest that GPT2 alters the distribution of metabolites between compartments and that this plays an essential role in allowing the cell to interpret environmental signals.

## Abstract 2

Acclimation to changes in the environment are crucial to optimising plant growth and productivity. In *Arabidopsis thaliana*, the glucose‐6‐phosphate/phosphate translocator GPT2 plays an important role in the acclimation to increased irradiance. Here we show that expression of GPT2 influences the partitioning of carbon between different storage pools, enhancing starch synthesis. Acclimation of metabolism occurs but is distinct in plants expressing or lacking GPT2.y

## Introduction

Plants in nature experience fluctuations in environmental conditions occurring on timescales ranging from seconds to months. In the very short term, changes in light intensity can give rise to large fluctuations in the redox poise of photosynthetic electron transport and in the concentrations of primary metabolites, which are buffered by rapidly induced regulatory processes (Bailey *et al*. 2001; Wulff‐Zottele *et al*. 2010; Dietz & Pfannschmidt 2011). In the longer term, changes in weather patterns can give rise to sustained changes in ambient light (e.g. transition from cloudy to sunny weather). As sessile organisms, plants must respond biochemically to long‐term changes in conditions in order to maintain efficient levels of photosynthesis and avoid stress (Takahashi & Badger 2011).

Variations in light intensity directly affect photosynthesis by changing the amount of energy available for electron transport and carbon fixation. Photosynthetic acclimation allows plants to respond to changes in light intensity, optimizing the balance of light absorption and use, reducing susceptibility to stress (Walters 2005). When plants are subjected to increased light, acclimation can result in sustained increases in photosynthetic capacity (e.g. Murchie *et al*. 2002; Oguchi *et al*. 2003; Athanasiou *et al*. 2010).

During development, photosynthetic acclimation can be achieved by altering plant's morphology; for example, by altering leaf area and thickness and altering root/shoot ratios (Murchie & Horton 1997; Ballaré 1999; Weston *et al*. 2000; Walters 2005). In mature tissues by contrast, the responses of photosynthesis to changes in light intensity must be achieved dynamically, via changes in protein concentrations in pre‐existing cells (Leister 2003; Li *et al*. 2009). We previously demonstrated that developmental and dynamic acclimation are, to some extent, distinct processes in *Arabidopsis thaliana* (Athanasiou *et al*. 2010).

When a mature plant is exposed to increased light, a multitude of potential signals is generated, which must be sensed and interpreted in a way that gives rise to an increase in the capacity for photosynthesis (for a review, see Hausler *et al*., 2014). Factors known to change gene expression include signals from light receptors, the redox poise of cellular components and the concentrations of metabolites, in particular various sugars. The cell is capable of detecting this complex range of signals and distinguishing these from other perturbations that have comparable effects on some or all of the signals.

Under conditions where mature, fully expanded leaves are subjected to a sustained increase in light intensity, *Arabidopsis* plants are typically capable of substantially increasing their photosynthetic capacity (*P*_max_; Athanasiou *et al*. 2010). *Arabidopsis* accession Wassilewskija‐4 (Ws‐4) shows a strong dynamic acclimation of photosynthetic capacity, increasing *P*_max_ by up to 40% when moved from a low to a higher irradiance and this ability to acclimate dynamically plays a major role in determining plant's fitness in naturally fluctuating environments (Athanasiou *et al*. 2010). Regulation of acclimation to high light (HL) has been suggested to involve redox signals from electron transport, changes in carbohydrate/nutrient status and hormone levels, and requires communication between the chloroplast, cytosol and nucleus (retrograde and anterograde signalling) (Hausler *et al*. 2014). The exact signalling mechanisms giving rise to these increases in *P*_max_ remain unknown (for a review, see Walters 2005). We previously showed that a gene (*At1g61800*) encoding a glucose 6‐phosphate/phosphate translocator GPT2 is required for acclimation of mature leaves to an increase in light; in a T‐DNA insertion knockout of *GPT2* (*gpt2.2*), increases in light intensity do not lead to substantial increases in *P*_max_ following a fourfold increase in light from 100 to 400 *μ*mol m^−2^ s^−1^ (Athanasiou *et al*. 2010). The non‐acclimating *gpt2.2* line is therefore a useful tool to study the dynamic acclimation response in *Arabidopsis*. In addition, the commonly used accession Columbia (Col‐0) has a similar phenotype, in terms of acclimation to increased light, to the *gpt2.2* mutant and to the corresponding mutant, *gpt2.1*, in a Col‐0 background.

There are 16 plastidic phosphate translocator genes found in *Arabidopsis*, six of which encode functional genes (Knappe *et al*. 2003; Fischer 2011). These function as antiporters, swapping inorganic phosphate and phosphorylated C_3_, C_5_ and C_6_ compounds across the plastid envelope in a strictly 1:1 exchange (Flügge 1999). Among the characterized translocators, the two GPT proteins are the least substrate specific, although glucose 6‐phosphate (G6P) is the favoured substrate (Kammerer *et al*. 1998; Eicks *et al*. 2002). In heterotrophic tissues, it has been proposed that GPT proteins (primarily GPT1) take G6P into plastids for incorporation into starch or to feed the oxidative pentose phosphate pathway (Niewiadomski *et al*. 2005; Kunz *et al*. 2010). *GPT1* is essential for the growth and development of *Arabidopsis*; homozygous T‐DNA insertion knockouts of GPT1 are gametophyte lethal (Niewiadomski *et al*. 2005). *GPT2* knockout plants are viable and show phenotypically normal growth under standard laboratory growth conditions. Only under variable growth conditions has a clear growth phenotype been demonstrated (Athanasiou *et al*. 2010).

Expression of *GPT2* can be induced under a range of treatments and at various developmental stages, as well as in a number of mutants impaired in carbon metabolism or its regulation. In wild‐type plants, *GPT2* expression is induced in imbibed seeds (Finch‐Savage *et al*. 2007), in response to exogenous and endogenous increases in sucrose (Lloyd & Zakhleniuk 2004; Gonzali *et al*. 2006), in response to an increase in light intensity (Athanasiou *et al*. 2010) and during senescence (Pourtau *et al*. 2006). Taken together, these results are consistent with GPT2 expression being induced in response to an accumulation of sugars in the cell.

To better elucidate processes occurring during acclimation to increased light and how GPT2 affects those processes, we have examined changes in metabolite levels in wild‐type and *gpt2* plants, concentrating on the regulatory events occurring at an early stage in acclimation to increased light, when GPT2 expression is maximal. We show that metabolite pools in both wild‐type and *gpt2* plants are altered in response to HL, even though these lines differ in their ability to increase photosynthetic capacity. The metabolic signatures of this acclimation differ between plants, implying that the responses observed are mechanistically different. We suggest that the partitioning of G6P between the chloroplast and the cytosol may provide a signal that allows the plant to sense and respond to environmental changes.

## Materials and Methods

### Plant growth and tissue preparation

Seeds of *Arabidopsis thaliana* accession Wassilewskija‐4 (wild‐type) and a homozygote T‐DNA insertion knockout of the *GPT2* gene (*gpt2.2*, FLAG_326E03; INRA, Versailles, France) were used in all experiments. Selected measurements are also shown for Col‐0 and the *gpt2.1* mutant. Plants for acclimation experiments were grown with an 8 h day/16 h night cycle for 8 weeks at an irradiance of 100 *μ*mol m^−2^ s^−1^ white fluorescent light until mature. Plants were then transferred to an irradiance of 400 *μ*mol m^−2^ s^−1^ [high light (HL)] or left at 100 *μ*mol m^−2^ s^−1^ [low light (LL)] as a control. Both wild‐type and *gpt2* seeds were generated at the same time from plants grown under our LL conditions throughout their life cycle. For photosynthetic measurements, the *in situ* rate of net CO_2_ fixation was measured on fully expanded leaves using a CIRAS 1 infrared gas analyser (PP Systems, Amesbury, MA, USA) under ambient light and CO_2_ conditions at the end of the photoperiod. A two‐way anova was performed on the data to determine significant differences (*P* < 0.05).

### Microarray analysis

Microarray analyses were carried out as in Athanasiou *et al*. (2010). At each time point, a mature leaf was detached and flash‐frozen in liquid N_2_. RNA extraction was carried out using an RNeasy mini kit (Qiagen, Venlo, the Netherlands). Total RNA was used to produce cDNA, which was then hybridized to an *Arabidopsis* (*Arabidopsis thaliana*) ATH1‐121501 oligonucleotide array (Affymetrix, St Clara, CA, USA) according to the manufacturer's instructions. The arrays were read by an Agilent GeneArray scanner 3000 7G using Affymetrix GCOS (GeneChipOperating Software) V1.4. Quality control of the arrays was carried out to check for outliers using dChip software (Li & Wong 2001). The normalization and expression analysis was performed using robust multichip averaging (Bolstad *et al*. 2003). Differential expression was assessed with a modified *t*‐test on logarithmically scaled data using Cyber‐T (Baldi & Long 2001). Differentially expressed genes were identified using the criteria of Cyber‐T *P*‐values less than 0.01, mean expression level greater than 100 in at least one condition and mean fold change greater than 2. Gene annotation was derived from The Arabidopsis Information Resource (release 10.0; http://www.arabidopsis.org/). The Affymetrix chip analysis was performed at The Bioinformatics Core Facility (BCF) of the University of Manchester (Manchester, UK).

### Fourier Transform Infrared (FTIR) spectroscopy

All chemicals, reagents and solvents used were purchased from Sigma‐Aldrich (St Louis, MO, USA). For FTIR spectroscopy analysis, fully expanded leaves were excised under growth light conditions using a razor blade, flash‐frozen and lyophilized overnight, after which the leaves were ground and transferred to −80 °C storage prior to extraction. Leaf tissue [30 mg dry weight (DW)] was homogenized in 1.5 mL of HPLC‐grade water, 15 *μ*L of homogenate was directly loaded on to the wells of a silicon 96‐well IR target plate (Bruker, Coventry, UK) and dried.

Sample homogenates were loaded onto the IR target plate in duplicate and two readings were taken from each sample well to serve as analytical replicates. The plate was loaded onto a motorized high‐throughput stage (HTR‐XT; Bruker, Billerica, MA, USA) attached to a Bruker Equinox 55 FTIR and run in transmission mode according to Winder *et al*. (2004).

### Gas chromatography electron impact time‐of‐flight mass spectrometry (GC‐EI‐TOF/MS) analysis

All chemicals, reagents and solvents used were purchased from Sigma‐Aldrich. Fully expanded leaves were excised using a razor blade, flash‐frozen and lyophilized overnight, ground and transferred to a −80 °C storage prior to extraction. Tissue aliquots (30 mg DW) were taken for the GC‐EI‐TOF/MS extraction procedure, which precisely followed that of Allwood *et al*. (2009) and Lisec *et al*. (2006). Polar phase extracts were dried using a centrifugal vacuum concentrator and spiked with 100 *μ*L of an internal standard solution containing 200 *μ*g mL^−1^ succinic‐d4 acid, glycine‐d5 and malonic‐d2 acid before analysis. Quality control samples were prepared by mixing equal volumes of the upper polar phase of all of the sample extracts, and these were analysed throughout the gas chromatography–mass spectrometry (GC‐MS) analyses so that analytical reproducibility could be assessed, as detailed in Dunn *et al*. (2011).

Because many of the metabolites within central metabolism are non‐volatile, a two‐step chemical derivatization was applied following the method detailed in Allwood *et al*. (2009). Samples were analysed within a single randomized analytical block. Quality control samples were interspersed throughout the block, permitting the assessment of instrument stability and removal of irreproducible metabolite features. Analyses were carried out with a Leco Pegasus III (4D) GC × GC/MS in GC/MS mode (Leco Corp., St. Joseph, MO, USA), with a Gerstel MPS‐2 autosampler (Gerstel, Baltimore, MD, USA) and an Agilent 6890N gas chromatograph (Agilent Technologies, Santa Clara, CA, USA), according to the method of Begley *et al*. (2009).

Raw GC‐EI‐TOF/MS data were baseline‐corrected, aligned and subjected to deconvolution to produce an XY matrix suitable for statistical analyses, following the method of Begley *et al*. (2009). Peak identities were putatively assigned on the basis of mass spectral similarity to NIST library and Golm Metabolome Database entries (Kopka *et al*. 2005) or were unambiguously assigned by mass spectral and retention index matching with an in‐house library (Begley *et al*. 2009) from the Manchester Metabolomics Database (Brown *et al*. 2009). Metabolite identifications were considered unambiguous when they had a match score of >800 and retention index within 20 units.

### Statistical analysis of FTIR and GC‐MS data

The calculated FTIR absorbance spectra were exported directly into Matlab R2008 (The MathWorks Inc., Natwick, MA, USA). Pre‐processing of the FTIR data involved five‐point smoothing by the Savitzky–Golay algorithm (Savitzky & Golay 1964). For GC‐MS, after deconvolution, the metabolite and metabolite level pairs were imported into Matlab. Post pre‐processing, the FTIR or GC‐MS‐derived XY outputs were independently examined using principal component analysis (PCA) as described in Allwood *et al*. (2006). Further, based upon the PCs generated by PCA, a supervised multivariate technique known as PC‐discriminant function analysis (PC‐DFA) was used to discriminate between the experimental groups based on an *a priori* knowledge of experimental class structure (Goodacre *et al*. 1998, 2004). PC‐DFA were generated and validated following the methods of Kaderbhai *et al*. (2003) and Allwood *et al*. (2006). N‐way anova was also performed to identify variables that were significant at a 99.9% confidence limit.

### Anion‐exchange liquid chromatography–tandem mass spectrometry (LC‐MS/MS)

Mature leaf tissue was excised 8, 56 or 104 h after first exposure to HL. Control (LL) samples were excised after 8 h. Tissue was flash‐frozen in liquid N_2_ and stored on dry ice until used. Frozen tissue powder was extracted using chloroform–methanol as described in Lunn *et al*. (2006). Phosphorylated intermediates and organic acids were quantified by high‐performance anion‐exchange chromatography coupled to tandem mass spectrometry, operating in negative ion mode, as described in Lunn *et al*. (2006). Metabolites were quantified by comparison against authentic standards. Measurements of T6P were corrected for ion suppression using a deuterated‐T6P internal standard. Technical replicates were averaged and a two‐way anova was performed on the data (*P* < 0.05) using SPSS (IBM, Armonk, NY, USA).

### Sucrose and starch analyses

Enzymatic assays were carried out on extracts from three fully expanded leaves from three separate plants. Tissue was excised and flash‐frozen in liquid nitrogen every 2–4 h throughout the photoperiod and stored at −80 °C before analysis. Starch was estimated using a total starch assay (Megazyme, Wicklow, Eire, Method E). Sucrose measurements were collected using a sucrose assay kit (Sigma, Poole, UK). Three biological replicates were assayed at each time point and each measurement was replicated three times. Technical replicates were averaged and a two‐way anova was performed on the data (*P* < 0.05) using SPSS (IBM).

## Results

### Loss of acclimation results in a loss of photosynthesis under growth conditions

**Figure 1:**
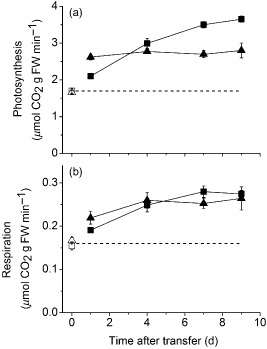
Photosynthetic rates and respiration during acclimation. (a) Photosynthetic rate at the end of the photoperiod measured on attached leaves of plants within growth cabinets. (b) Dark respiration measured at the end of the photoperiod within growth cabinets. Plants of Ws‐4 wild‐type (squares) and *gpt2.2* (triangles) lines were grown at low light (LL, 100 *μ*mol m^−2^ s^−1^ white light, 8 h light, 20 °C/16 h dark, 18 °C) for 8 weeks. They were then transferred to high light (HL, 400 *μ*mol m^−2^ s^−1^ white light, 8 h light, 20 °C/16 h dark, 18 °C; closed symbols) or maintained at LL (open symbols). LL measurements were made at the beginning of the acclimation period for comparison. Points represent the mean of at least three replicates from three separate plants. Error bars represent ±1 SE. Significant differences between samples were determined using a two‐way anova (*P* < 0.05, see the Results section for details).

Previously, we showed that the photosynthetic capacity of wild‐type Ws‐4 plants increases significantly (∼40%) over a 7‐day‐acclimation period following a transfer from 100 (LL) to 400 (HL) *μ*mol m^−2^ s^−1^ irradiance, and that this response requires GPT2 (Athanasiou *et al*. 2010). In order to determine the impact of this on plant's performance under actual growth conditions, measurements of photosynthesis and respiration were made on intact plants in growth chambers at ambient light and CO_2_ (Fig. 1). At LL, there was no significant difference in the rate of CO_2_ assimilation between wild‐type and *gpt2.2* plants. Higher irradiance resulted in an immediate increase in the rate of photosynthesis (Fig. 1a) in both wild‐type and *gpt2.2* plants; however, the proportional increase was smaller than the proportional increase in irradiance, indicating a degree of saturation of photosynthesis. This initial increase was greater in *gpt2.2* than it was in wild type, reflecting the fact that there is a higher *P*_max_ in these plants when grown in LL (Athanasiou *et al*. 2010). Over subsequent days, however, photosynthetic rates increased significantly in wild‐type plants, whereas no further increase was observed in *gpt2.2* plants. This change reflected the differences in *P*_max_ observed over a 7‐day‐acclimation period in the different plants (Athanasiou *et al*. 2010). Respiration also increased (Fig. 1b), with significantly higher rates observed in both wild‐type and *gpt2.2* plants by the end of the first day. This increase was sustained over subsequent days, with no significant difference between the two lines. Measurements of *in situ* gas exchange were also performed on plants of Col‐0 and of *gpt2.1*, a *GPT2* insertion mutant in the Col‐0 background (Supporting Information Fig. S1). As expected from previous measurements (Athanasiou *et al*. 2010), neither of these lines were able to acclimate their photosynthetic capacity in response to an increase in irradiance in mature leaves. Consistent with this, the actual rate of photosynthesis under growth conditions also did not increase following an increase in growth irradiance. No significant differences were observed in gas exchange parameters between Col‐0 and *gpt2.1*. Based upon the above results, we conclude that net carbon fixation is greater at HL, compared to LL, in both wild‐type and *gpt2* plants but that the lines differ from one another as acclimation proceeds.

### Gene expression patterns in response to HL differ in wild‐type and *gpt2.2* plants

To examine the impact of loss of GPT2 on overall gene expression, global transcriptome analysis was performed on leaf tissue at LL and after 4 h of HL from *gpt2.2* and Ws‐4 wild‐type plants. A comparison of the two lines at LL showed significant differences in the transcript levels for a number of genes, indicating that the low level of GPT2 expression observed under LL (i.e. non‐stressed) conditions still has an impact on transcription.

Using MapMan transcriptomic software, we compared transcription in the two lines under LL conditions. A number of metabolic bins (Thimm *et al*. 2004) were highlighted as showing large transcript level differences (Supporting Information Fig. S2) including trehalose metabolism (with TPS‐8, TPS‐10 and TPS‐11 overexpressed in LL *gpt2.2* plants compared with LL wild type) and lipid metabolism (with genes overexpressed in *gpt2.2* plants). Bins containing genes for raffinose, starch and sucrose metabolism also showed differences, with genes both up‐ and down‐regulated in these categories.

Notably, there were striking differences in genes associated with photosynthetic light reactions; after applying stringent criteria to gene expression levels (*P* < 0.01, fold change above 1.5 and expression levels over 100 in at least one condition, Table 1) 26 out of 111 genes (23%) were significantly different between the two lines, the vast majority (24) of these being greater in *gpt2.2* plants. Of these overexpressed light‐reaction genes, 17 are chloroplast‐encoded, which is representative of a general trend in *gpt2.2* plants; 36 of 77 chloroplast‐encoded genes identified by MapMan software (47%) are significantly higher in *gpt2.2* plants under LL control conditions, with only one being significantly lower (Supporting Information Table S1).

**Table 1:** Fold changes in gene expression

ID	Gene ID	Description	*gpt2.2* LL versus Ws‐4 LL	*gpt2.2* HL versus *gpt2.2* LL	*Ws‐4* HL versus Ws‐4 LL
244977_at	atcg00730	PETD, subunit of cytochrome b6/f	9.147	1.226	1.448
245007_at	atcg00350	PSAA, PSI reaction centre	8.773	−1.05	2.282
245047_at	atcg00020	PSBA, chlorophyll binding protein D1	6.765	1.125	1.045
244932_at	atcg01060	PSAC, subunit of PS I	4.241	1.035	1.480
245002_at	atcg00270	PSBD, PSII D2 protein	3.77	1.294	1.495
244973_at	atcg00690	PSBT, PS II 5 kD protein subunit PSII‐T.	3.002	1.212	1.194
244972_at	atcg00680	PSBB, CP47, subunit of PSII	2.712	1.46	1.127
259970_at	at1g76570	chlorophyll A‐B binding family protein	2.404	1.178	1.949
245021_at	atcg00550	PSBJ, PSII component	2.317	1.05	−1.039
260481_at	at1g10960	ATFD1, ferredoxin 1	2.308	−1.08	1.723
244964_at	atcg00580	PSBE, PSII cytochrome b559.	2.195	1.221	−1.05
244995_at	atcg00150	ATPI, subunit of ATPase complex CF0	2.028	−1.484	1.191
245025_at	atcg00130	ATPF, ATPase F subunit.	1.843	−1.15	−1.045
253790_at	at4g28660	PSB28, PS II reaction center	1.791	−1.077	1.875
265722_at	at2g40100	LHCB4.3, light harvesting complex PSII	1.781	−1.357	1.736
245026_at	atcg00140	ATPH, ATPase III subunit	1.682	−1.019	1.129
244975_at	atcg00710	PSBH, 8kD component of PSII	1.649	1.138	1.258
256015_at	at1g19150	LHCA6	1.611	−1.801	−1.451
244963_at	atcg00570	PSBF, PSII cytochrome b559	1.565	1.137	−1.033
251701_at	at3g56650	thylakoid lumenal 20 kDa protein	1.559	−1.592	−1.317
254398_at	at4g21280	PSBQ, oxygen‐evolving enhancer protein	1.538	1.368	−1.297
264959_at	at1g77090	thylakoid lumenal 29.8 kDa protein	1.531	−1.081	1.204
245003_at	atcg00280	PSBC, CP43 subunit of PSII	1.521	−1.036	−1.011
258993_at	at3g08940	LHCB4.2, light harvesting complex PSII	1.52	−2.479	−3.386
245368_at	at4g15510	PSII reaction center PsbP family protein	1.496	−1.908	−1.547
258239_at	at3g27690	LHCB2.4, light harvesting complex PSII	1.403	−2.607	−2.886
259491_at	at1g15820	LHCB6, CP24	1.36	−1.329	−1.64
251784_at	at3g55330	PPL1, PsbP‐like protein	1.312	−1.706	−1.234
267569_at	at2g30790	PSBP‐2, PS II subunit P‐2	1.131	−1.565	−1.371
261388_at	at1g05385	PS II 11 kDa protein‐related	1.082	−1.64	−1.363
263114_at	at1g03130	PSAD‐2 , PS I subunit D‐2	1.067	−2.29	−2.837
244966_at	atcg00600	PETG, Cytochrome b6‐f subunit V	1.054	−4.754	1.266
261769_at	at1g76100	PETE1, plastocyanin 1	−1.022	−1.805	−2.485
244974_at	atcg00700	PSBN, PSII low MW protein	−1.1	−1.607	1.503
252130_at	at3g50820	PSBO2, PSII subunit O‐2	−1.232	−1.572	−1.66
265149_at	at1g51400	PS II 5 kD protein	−1.272	−3.062	−2.758
245000_at	atcg00210	YCF6	−1.556	1.856	1.059
245024_at	atcg00120	ATPA, ATPase alpha subunit	−1.584	−1.563	−1.062

Genes associated with the light reactions were mined using MapMan software. Of 111 genes, those which changed significantly between Ws‐4 and *gpt2.2* at LL, or in either line during HL acclimation, are shown here. Significantly changed genes must show a fold change of more than 1.5‐fold.

HL, high light; LL, low light.

In contrast, when comparing LL and HL plants using MapMan software, changes in genes linked to the photosynthetic light reactions were conspicuously absent (Supporting Information Figs S3 & S4). Despite there being a significant increase in both *P*_max_ (Athanasiou *et al*. 2010) and photosynthetic rate under growth conditions (Fig. 1), only 14 of 111 genes (12%; Table 1; Supporting Information Table S1) associated with the light reactions were significantly altered in wild‐type plants in response to HL. In *gpt2.2* plants, only 16 of these genes are significantly changed. During *developmental* acclimation to HL, gene expression is regulated in response to changes in the photosynthetic pathway (Pfannschmidt 2003). However, previous work has shown a lack of correlation between photosynthesis and gene expression at different intensities of steady‐state light (Walters 2005; Piippo *et al*. 2006). In this experiment, photosynthetic gene expression does not change substantially after transfer to HL in either wild‐type or *gpt2.2* plants. This suggests that changes in transcription levels of genes encoding photosynthetic proteins are not necessary to increase photosynthetic capacity in response to increased light in the range examined here.

In wild‐type plants, changes are evident in MapMan bins for cell wall synthesis, modification and degradation, starch metabolism and trehalose metabolism upon transfer to HL (Supporting Information Fig. S3). There were fewer changes in *gpt2.2* plants, with fewer genes in the cell wall synthesis, cell wall modification and trehalose metabolism bins showing significant changes (Supporting Information Fig. S4). We observe fewer significant changes in gene expression in *gpt2.2* plants versus wild‐type plants (421 versus 666). There are 816 genes that show a significant change in either or both lines. Of these, 33% change similarly in both wild‐type and *gpt2.2* plants, highlighting a conserved response to increased light intensity (see Supporting Information Table S1 for genes differentially expressed between lines). However, 395 (48%) of these 816 significantly altered genes are changed in wild‐type plants only, showing that there is a clear response in the acclimating wild‐type line that is not observed in *gpt2.2* plants.

### Sucrose accumulation is independent of GPT2 expression during acclimation to HL, but starch accumulation is significantly lower in *gpt2.2* plants

**Figure 2:**
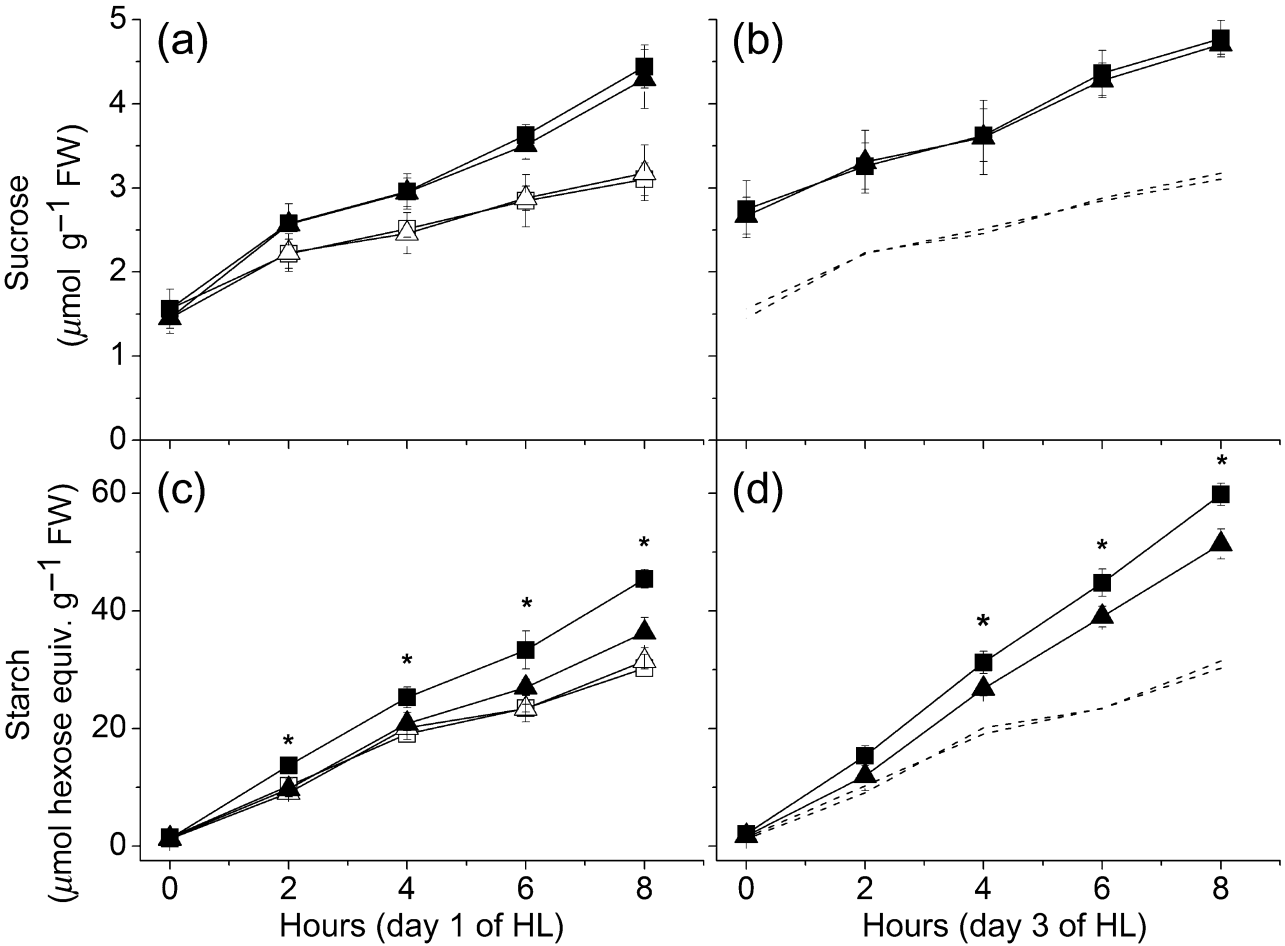
Sucrose and starch accumulation during acclimation. Plants of Ws‐4 wild type (squares) and *gpt2‐2* (triangles) were grown at low light (LL, 100 *μ*mol m^−2^ s^−1^ white light, 8 h light, 20 °C/16 h dark, 18 °C) for 8 weeks. They were then moved from low to high light (HL, 400 μmol m^−2^ s^−1^ white light, 8 h light, 20 °C/16 h dark, 18 °C; closed symbols) at day 0 or maintained at LL (open symbols). (a) Sucrose content measured in Ws‐4 wild‐type and *gpt2.2* plants on the first day after transfer to HL. (b) Sucrose content measured in Ws‐4 wild‐type and *gpt2.2* plants on the third day after transfer to HL. (c) Levels of starch measured in Ws‐4 wild‐type and *gpt2.2* plants on the first day after transfer to HL. (d) Levels of starch measured during acclimation in Ws‐4 wild‐type and *gpt2.2* plants on the third day after transfer to HL. Points represent the mean of at least five biological replicates. Error bars represent ±1 SE. Significant differences between samples were determined using a two‐way anova. Asterisks denote where levels in Ws‐4 wild‐type and *gpt2.2* plants under HL treatment differ significantly (*P* < 0.05).

Given the increase in the net rate of photosynthesis observed in response to HL (Fig. 1), changes in the rate of flux of carbon compounds into different storage pools were to be expected. There are a number of temporary leaf carbon stores, the most prominent being sucrose and starch, both of which are synthesized from triose phosphates produced during photosynthesis. Levels of starch and sucrose were measured in Ws‐4 wild‐type and *gpt2.2* leaves through the first and third photoperiods after transfer to HL. Both starch and sucrose levels within leaf tissue exhibit a diurnal cycle of accumulation during the day and degradation at night under both LL and HL conditions (Fig. 2 and data not shown).

Accelerated net accumulation of sucrose occurred in both lines on the first day of HL treatment, with leaf sucrose content being significantly increased by 2 h of exposure to HL compared with LL, and this higher rate of sucrose accumulation was maintained throughout the photoperiod (Fig. 2a). Overnight, this sucrose pool was not completely lost from the leaf, so that at dawn on day 3, there was significantly more sucrose in HL‐grown plants than in those maintained at LL (Fig. 2b). Unlike on day 1 of HL, the rate of accumulation of sucrose through the photoperiod was similar in both HL and LL conditions on day 3. Notably, no differences were observed between sucrose levels in wild‐type and *gpt2.2* plants, either in the rate of accumulation in the light or of the nighttime loss of sucrose from the leaf, in spite of the differing rates of photosynthesis in these plants. This suggests that rates of synthesis and export of sucrose are carefully regulated such that the leaf pools are not affected in response to increased irradiance, except on the first day, when there is a net accumulation of an additional sucrose pool, which is then maintained on subsequent days.

In contrast to sucrose, there were significant differences in the pattern of starch accumulation between Ws‐4 wild‐type and *gpt2.2* plants (Fig. 2c,d). In wild‐type plants, there was a rapidly induced increase in the rate of starch accumulation after transfer to HL, with a significant rise in starch already being apparent after 2 h of HL and a faster rate of starch accumulation being maintained throughout the photoperiod (Fig. 2c). In contrast, in *gpt2.2* plants, there was no detectable increase in starch accumulation in response to HL in the early part of the photoperiod (0–4 h) with significant differences only occurring towards the end of the day. This was in spite of the higher rate of photosynthesis in *gpt2.2* plants in the first photoperiod of HL (Fig. 1).

On day 3, the rate of starch accumulation was higher in HL than on day 1, in both wild‐type and *gpt2.2* leaves, implying tha an acclimation of starch synthesis had occurred. However, wild‐type plants accumulated significantly higher amounts of starch than *gpt2.2* throughout the HL photoperiod.

### Global metabolite analyses show distinct acclimation of metabolism in wild‐type and *gpt2* plants

By the end of the first photoperiod of HL treatment, *gpt2.2* plants accumulated similar levels of sucrose as that of Ws‐4 wild type but significantly lower levels of starch (Fig. 2), despite showing a higher rate of photosynthesis on that day (Fig. 1). This suggests that plants without GPT2 might be diverting carbon into other pools, possibly including lipids, proteins and cell walls. In order to elucidate processes occurring during HL acclimation and to examine the consequences of these differences, global changes in metabolites were examined in both wild‐type and non‐acclimating *gpt2.2* lines. FTIR spectroscopy was used for generating metabolic fingerprints and GC‐MS for non‐targeted metabolite profiling.

**Figure 3:**
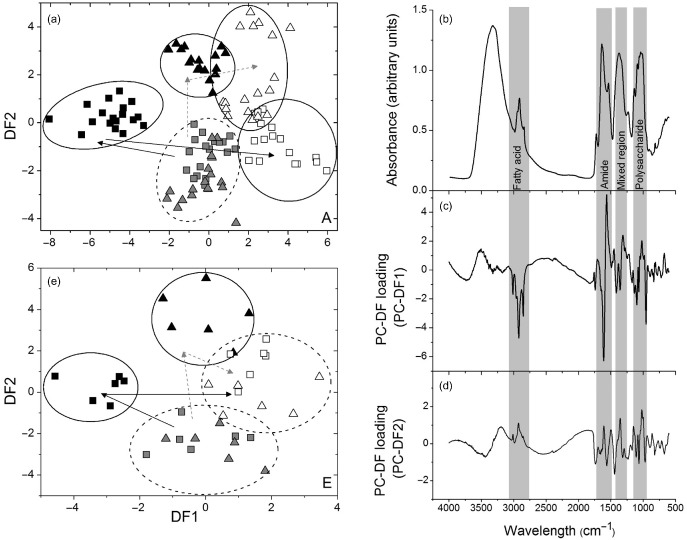
Fourier transform infrared (FTIR) and gas chromatography–mass spectrometry (GC‐MS) analysis of changes in metabolism during acclimation to high light (HL). (a) Principal component‐discriminant function analysis (PC‐DFA) model of FTIR spectra of extracts from Ws‐4 wild‐type and *gpt2.2* lines in the first 3 d after transfer to HL conditions. Circles and arrows are indicative of clustering only and have no statistical significance. Plants were grown for 8 weeks at low light (LL, 100 *μ*mol m ^−2^ s^−1^) and then transferred to HL (400 *μ*mol m^−2^ s^−1^) for up to 3 d. Ten PCs were used in the calculation, accounting for 99.1% of the total variance. PC1 explained 56.2% of the total variance, with PC2 explaining a further 24% of the total variance. Grey symbols represent plants at the end of day in LL, black symbols represent plants at the end of 1 photoperiod of HL. Open symbols represent plants at the end of 3 d of HL treatment. (b) A representative FTIR spectrum of an extract from Ws‐4 wild‐type plants grown under LL conditions. (c–d) PC‐DF loadings highlighting parts of the FTIR spectrum where a chemical functional group exhibits a particular absorbance important for the PC‐DFA model. PC‐DFA loading vectors are plotted against wavenumbers (cm^−1^) for (c) PC‐DF1 and (d) PC‐DF2. (e) PC‐DFA model constructed from the GC‐MS data from extracts from Ws‐4 wild‐type and *gpt2.2* lines in the first 3 d after transfer to HL conditions. PC1 accounted for 65% of the total variance, with PC2 accounting for a further 28%. Samples were collected at the end of the photoperiod in plants maintained at LL (grey symbols) and transferred to HL for 1 d (black symbols) and 3 d (open symbols).

Fully expanded leaves were collected at the end of the first and third photoperiods from HL‐treated plants and from controls maintained at LL. PCA was performed on the FTIR data, and PC‐DFA, a supervised multivariate technique, was applied to the principal components to identify the responses of each plant type to HL (Fig. 3a). Data from wild‐type and *gpt2.2* LL samples were weakly discriminated by PCA but diverge when subjected to HL treatment (data not shown). In wild‐type plants, the metabolic changes are substantial, and the variance between the days of the HL treatment can be mostly accounted for by PC‐DF loading 1. The changes in *gpt2.2* plants are smaller, with the variance accounted for by both PC‐DF loadings 1 and 2. The most substantial changes in metabolites occur on the first day of acclimation in both Ws‐4 wild‐type and *gpt2.2* plants, presumably reflecting the direct effects of increased carbon fixation. On day 3 of the HL treatment, Ws‐4 wild‐type and *gpt2.2* samples tended to converge on the PC‐DFA plot, suggesting that the main metabolic differences between the lines occur in the early stages of acclimation (Fig. 3a). In terms of the variance between LL controls and day 1 samples, changes in wild‐type metabolism are mainly accounted for by PC‐DF1, and changes in *gpt2.2* plants mainly by PC‐DF2, which suggests that there were larger metabolic changes in Ws‐4 wild‐type than *gpt2.2*. PC‐DF loading plots allow us to examine which types of phenotypic changes underlie the variance between PC‐DFAs and therefore broadly between the two lines on the first day of acclimation (Fig. 3b–d). Loadings for both PC‐DFAs show changes in the fatty acid and polysaccharide regions of the IR spectrum, with substantial changes in the amide region. This suggests significant changes in whole leaf metabolism occurring in both wild‐type and non‐acclimating *gpt2.2* plants, with the responses being different in the two lines.

PC‐DFA was also carried out on data acquired by GC‐MS. GC‐MS was used to identify 85 polar compounds, with 39 of these being of unknown chemical structure (for full data set, see Supporting Information Table S2). All of the GC‐MS data were used in the PC‐DFA (Fig. 3e). As for the FTIR, there was poor separation of data for LL‐grown plants. When plants were exposed to HL for 1 photoperiod, a clear separation of wild‐type and *gpt2.2* plants could be observed, with PC‐DF1 explaining most of the change in wild‐type and PC‐DF2 being responsible for the differences in the mutant. By the third day of acclimation, a convergence of the data was observed, with the wild‐type and *gpt2.2* plants no longer being well discriminated. Both the FTIR and the GC‐MS data sets are highly reproducible between technical, analytical and biological replicates within the same experimental classes.

FTIR and GC‐MS analysis were also performed on plants of Col‐0 wild‐type and *gpt2.1* (Supporting Information Fig. S5). In contrast to the results comparing Ws‐4 and *gpt2.1*, no clear discrimination was observed between Col‐0 and the corresponding mutant. This is consistent with the data from other groups on the *gpt2.1* mutant and indicates that the major changes in metabolism separating Ws‐4 from *gpt2.2* are likely a consequence of different acclimation process occurring in those plants rather than a direct consequence of GPT2 expression.

### Targeted analysis of metabolites involved in starch and sucrose synthesis

Given that GPT2 is a G6P translocator, it is likely that expression of this protein will have a direct impact on the pools of metabolites involved in the pathways of sucrose and starch biosynthesis. To quantify these metabolites, targeted LC‐MS/MS was carried out on leaf samples collected at the end of the first, third and fifth photoperiods from plants exposed to HL and from controls maintained at LL. Twenty metabolites were identified by LC‐MS/MS on the first day of acclimation (Table 1; for the full LC‐MS/MS data set, see Supporting Information Table S3).

In plants maintained at LL, the majority of measured metabolites did not differ significantly between Ws‐4 wild‐type and *gpt2.2* plants. A notable exception to this was trehalose 6‐phosphate (T6P), with *gpt2.2* plants having over twice as much as wild type. T6P has previously been suggested to play a role in regulating photosynthetic capacity (Delatte *et al*. 2011). The concentration of shikimate was also found to be substantially higher in *gpt2.2* leaves than in Ws‐4 wild type under all treatments, while the concentration of succinate was lower.

When plants were transferred to HL, two major responses could be observed among the identified metabolites. Metabolites involved in flux from photosynthesis to either starch or sucrose typically increased upon first transfer to HL, but then decreased again as acclimation proceeded. This pattern of behaviour was notably observed for phospho*enol*pyruvate (PEP). Meanwhile, pyruvate and various intermediates of the TCA cycle exhibited a drop in concentration, which was then reversed as plants acclimated (for an overview, see Fig. 5). These two broad responses were observed in both Ws‐4 wild‐type and *gpt2.2* plants. Although the responses were qualitatively similar, there were quantitative differences between the lines, with individual metabolites showing exaggerated responses in the mutant (tending to increase further and then decrease more).

**Figure 4:**
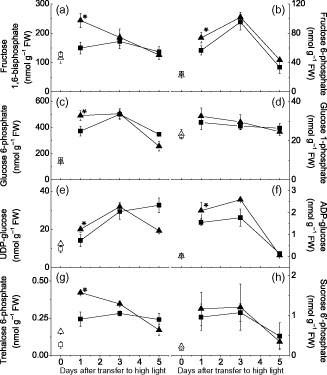
Sugar phosphate and sugar nucleotide levels during acclimation. Sugar phosphate and sugar nucleotide levels were measured using anion‐exchange liquid chromatography–tandem mass spectrometry (LC‐MS/MS) on tissue from plants at low light (LL) or transferred to high light (HL) or up to 5 d. Sugar phosphate/nucleotide levels were determined in plants grown for 8 weeks at LL (100 *μ*mol m^−2^ s^−1^, 8 h light, 20 °C/16 h dark, 18 °C) or grown at LL and then transferred to HL (400 *μ*mol m^−2^ s^−1^, 8 h light, 20 °C/16 h dark, 18 °C). (a) fructose 1,6‐bisphosphate, (b) fructose 6‐phosphate, (c) glucose 6‐phosphate, (d) glucose 1‐phosphate, (e) UDP‐glucose, (f) ADP‐glucose, (g) trehalose 6‐phosphate and (h) sucrose 6′‐phosphate. Points represent the mean of three to five biological replicates. Error bars represent ±1 SE. Asterisks indicate data points where wild‐type and *gpt2.2* plants differ significantly (based on a two‐way anova, *P* < 0.05).

There were however a number of notable differences between the plants. Seven metabolites directly involved in starch and/or sucrose metabolism were identified [glucose 1‐phosphate (G1P), glucose 6‐phosphate (G6P), fructose 1, 6‐bisphosphate (F16BP), fructose 6‐phosphate (F6P), sucrose 6′‐phosphate (S6P), ADP‐glucose and UDP glucose], in addition to T6P, which is an important sugar signalling metabolite (Lunn *et al*. 2006). In general, concentrations of these significantly and substantially increased in both Ws‐4 wild‐type and *gpt2.2* plants by the end of the first day of HL treatment; only levels of glucose 1‐phosphate (G1P) and UDP‐glucose in wild type did not change significantly. In addition, on the first day of HL treatment, levels of all eight of these metabolites were higher in the *gpt2.2* plants than in Ws‐4 wild type, significantly so for all except G1P and S6P (Fig. 4; Table 1). Over longer time periods, the majority of identified sugar phosphates/nucleotide sugars showed a similar pattern in their response to increased light, with increases followed by a decline towards the end of the acclimation period. G6P levels remained significantly elevated relative to LL levels after 5 d HL, with levels in both Ws‐4 wild‐type and *gpt2.2* plants being double as those observed in control plants after 5 d at HL (Fig. 4c).

**Figure 5:**
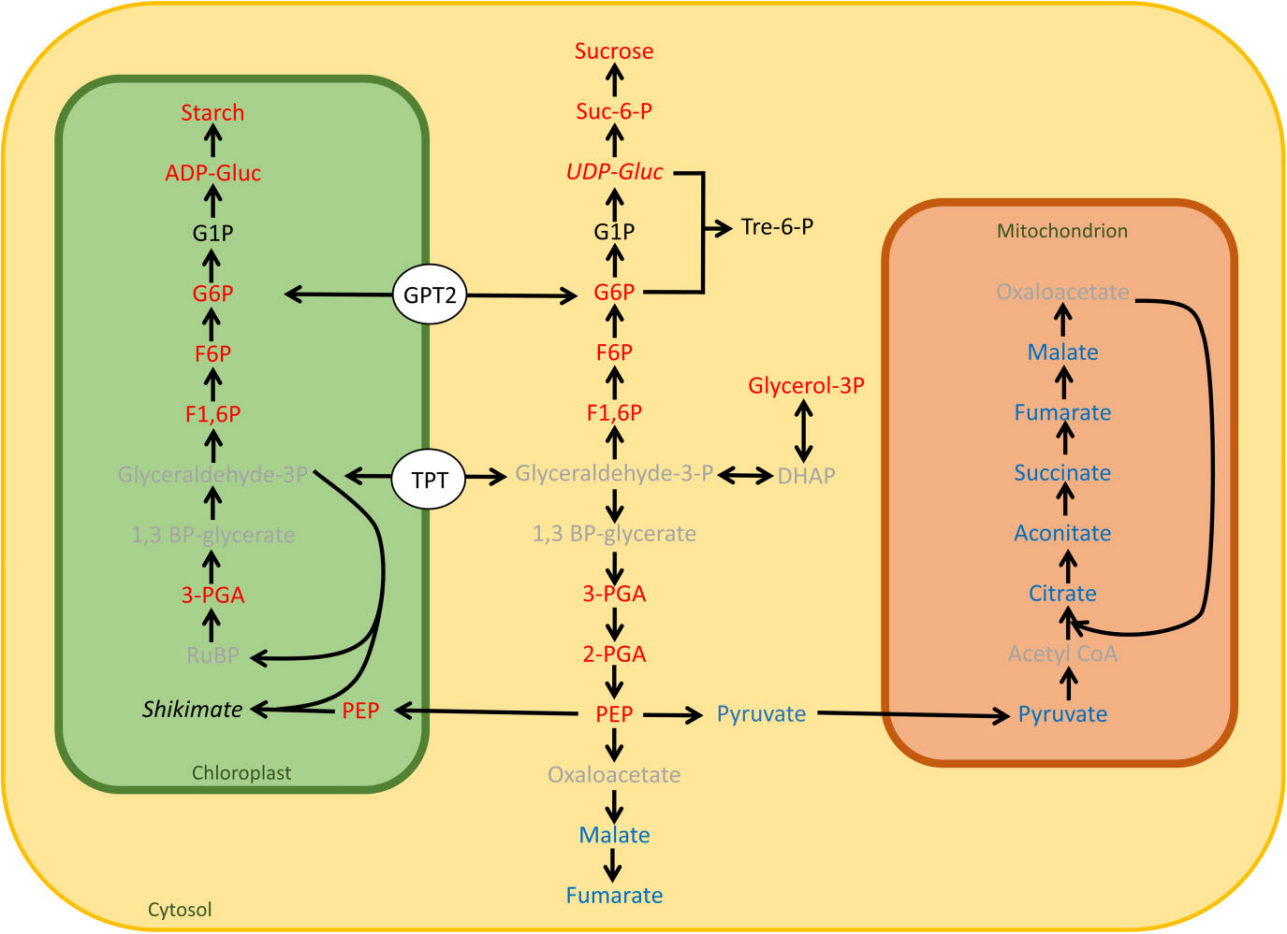
Summary of carbon metabolism changes occurring during acclimation to high light (HL). Increases in light result in rapid changes (within the first photoperiod) in concentrations of primary metabolites within the cell. Starch and sucrose levels increase rapidly (Fig. 3), as do a number of sugar phosphates and nucleotide sugars involved in their synthesis (Fig. 5). Triose phosphate is exported from the chloroplast primarily by the triose phosphate translocator (TPT) and can be channelled into sucrose synthesis via a number of intermediates, including G6P. GPT2 facilitates the movement of glucose 6‐phosphate (G6P) across the chloroplast envelope. It is proposed that the net direction of flux is into the chloroplast. G6P is a substrate for starch synthesis, accounting for the increased starch accumulation observed in wild‐type plants. Metabolites in red increase on day 1 of HL treatment, metabolites in blue decrease, relative to the LL control. Metabolites in black do not change. Metabolites in grey were not measured. In general, metabolites in red increase on day 1 and return to near control levels by day 7 in both Ws‐4 wild‐type and *gpt2.2* plants. Metabolites in italics show a different pattern in wild‐type and *gpt2.2* plants, and are further discussed in the text.

T6P levels are already significantly different between the two lines under LL control conditions; *gpt2.2* leaves had a significantly higher T6P content than wild type (Fig. 4g). T6P levels rose, relative to the LL controls, on the first day of HL in both plants; however, this effect was much more marked in *gpt2.2*. In the mutant line, T6P levels fell sharply through the acclimation period, falling to LL levels by day 5. In contrast, concentrations in wild type were substantially higher throughout the experiment in HL leaves and remained higher than LL controls at the end of the experiment. In contrast to most sugar phosphates, UDP‐glucose levels rose throughout the HL treatment. In *gpt2.2*, however, UDP‐glucose concentration fell upon longer exposure of HL, although it remained above the LL level at the end of the experiment. Qualitatively, the responses of PEP were similar to those of UDP‐glucose, with an elevated concentration being observed in the wild type but not in *gpt2.2* at the end of the acclimation period.

## Discussion

Previously, we demonstrated that the ability of plants to acclimate their photosynthetic apparatus to sustained changes in light has a substantial role in determining yield in naturally fluctuating conditions (Athanasiou *et al*. 2010). Here, we have shown that plants able to acclimate to higher light are also able to achieve a significantly higher rate of photosynthesis following acclimation (Fig. 1, Supporting Information Fig. S1). In addition, our results show that acclimation to light involves major changes in metabolism, which we suggest allow plants to buffer changes in their environment; increased irradiance results in a transiently altered metabolome; however, the tendency is for metabolites to tend towards starting levels after acclimation.

In plants grown at LL, there is little difference observed between Ws‐4 wild type and *gpt2.2* in terms of their physiology, with LL‐grown plants having similar growth, seed yield, photosynthetic/respiration rates and morphology (Athanasiou *et al*. 2010; Fig. 1). However, there *are* clear differences between the lines in terms of their transcriptomic profiles accompanied by weaker differences in FTIR analyses (Fig. 3), and GC‐MS and LC‐MS (Figs 3, 4). This discrimination is perhaps not surprising given that GPT2 expression is observed throughout plant development. This suggests that a lack of GPT2 expression has effects on transcription under control conditions despite *gpt2.2* plants having no obvious phenotype. Notably, there are differences in photosynthetic gene expression in *gpt2.2* plants, with higher transcript levels for genes linked to the light reactions and for most chloroplast‐encoded genes. *gpt2.2* plants have a slightly higher photosynthetic capacity at LL (Athanasiou *et al*. 2010) but nevertheless achieve the same photosynthetic rates at growth irradiance (Fig. 1). *gpt2.2* plants therefore have a different transcriptional strategy to achieve similar photosynthetic results. This points to a striking degree of homeostasis being achieved by these plants.

When plants are transferred from LL to HL, there is an immediate increase in the rate of CO_2_ fixation (Fig. 1), which is significantly greater in *gpt2.2* plants than in wild type. This is consistent with the observation that *gpt2.2* plants have a greater photosynthetic capacity when grown at LL (Athanasiou *et al*. 2010). When plants are exposed to HL, leaf concentrations of sucrose and starch increase in wild‐type plants. However, in spite of the higher rate of photosynthesis, net accumulation of sucrose in *gpt2.2* is identical and starch synthesis is lower than in wild type. Sucrose content of leaves is a product of rates of synthesis and export or breakdown. The lack of change of the diurnal accumulation of sucrose in response to increased photosynthesis suggests that these rates are carefully balanced. Nevertheless, several primary metabolites, including G6P, FBP and T6P, increase significantly and substantially more in *gpt2.2* than in Ws‐4 wild type. This indicates that plants without GPT2 are less able to buffer short‐term changes in carbohydrate metabolism.

There is evidence that the export of triose phosphates, via the triose phosphate translocator, is an important regulator of gene expression, especially at the early stages following a change in irradiance (Vogel *et al*. 2014). Mutants lacking TPT have previously been shown to be unable to acclimate to high irradiances (Walters *et al*. 2004. Rapid transient expression of transcription factors observed following an increase in irradiance is suppressed in the tpt2 mutant of Col‐0 (Vogel *et al*., 2014). This suggests that cytosolic concentrations of triose phosphates and/or other primary metabolites are important in regulating gene expression in a highly dynamic way. Our observation that GPT2 expression is important for the longer term responses to light is consistent with a role for GPT2 in modulating such effects. On the contrary, GPT2 has been shown to not be required for the sugar‐dependent rescue of the high chlorophyll fluorescence (HCF) phenotype observed in mutants deficient in both triose phosphate export and starch synthesis (Schmitz *et al*. 2012). This HCF phenotype seems to arise due to a mis‐assembly of the thylakoid membranes, with accumulation of disconnected light‐harvesting proteins (Schmitz *et al*. 2012), probably indicating an effect that is importantly already present under developmental conditions. Our previous data (Athanasiou *et al*. 2010) indicated that GPT2 was not important during developmental acclimation, consistent with the results from Schmitz *et al*. (2012). Nevertheless, the results here suggest that under both sets of conditions, the distribution of primary metabolites between chloroplast and cytosol is likely to be important.

The expression of GPT2 within the leaf will allow the exchange of G6P and inorganic phosphate (Pi) across the chloroplast envelope, with the direction determined by the relative concentrations of G6P and Pi. The first alternative is that G6P is exported from the chloroplast, with a concurrent import of Pi. Accumulation of phosphorylated intermediates following the increase in CO_2_ fixation will lower the levels of free Pi in both the cytosol and the chloroplasts. Low stromal levels of Pi lead to an inhibition of photosynthesis (Sharkey & Vanderveer 1989; Rao & Terry 1995). Export of G6P would supplement TP export, increasing cytosolic substrates for sucrose synthesis. At the same time, given the relatively low substrate specificity of GPT2, it could be acting to supplement TPT activity, exporting TPs. Phosphate released in sucrose synthesis would be reimported into the chloroplast. This scenario would lead to increases in sucrose synthesis due to increased cytosolic G6P and potentially to increases in photosynthetic rate due to an increased availability of Pi in the chloroplast. At the same time, carbon would be diverted away from starch synthesis. During the first 3 d of acclimation, plants without GPT2 maintain similar levels of net sucrose accumulation as that of wild type and have lower starch synthesis, while initially having a higher photosynthetic rate than wild‐type plants. Net export of sugar phosphates via GPT2 seems therefore unlikely, although without measurements of flux through the sucrose pool, this idea cannot be excluded.

The observation that starch accumulation is higher at HL in the wild type indicates that the net direction of G6P flux through GPT2 is more likely to be *into* the chloroplast. Fixed carbon exported as triose phosphate would be converted to hexose phosphate and then reimported into the plastid for incorporation into starch. By removing G6P from the cytosol, GPT2 may help buffer changes in carbon metabolism and divert more fixed carbon into starch. This model is consistent with the higher starch synthesis in wild type and also with observations from fractionation studies that G6P concentrations are higher in the cytosol than the chloroplast (Gerhardt *et al*. 1987; Vosloh 2011). The increase in starch synthesis in the wild type is rapid, with a significant increase in starch being detectable within 2 h of HL (Fig. 2), consistent with the rapid induction of GPT2 (20 fold increase within 2 h; Athanasiou *et al*. 2010).

Expression of GPT2 will allow equilibration of G6P and phosphate across the chloroplast envelope and will, we suggest, lower the cytosolic concentration of G6P. G6P has many direct and indirect effects on metabolism that have already been elucidated. G6P levels are known to positively regulate sucrose synthesis (Doelhert and Huber, 1983). In addition, while G6P is a direct allosteric activator of sucrose phosphate synthase (SPS), Pi is an allosteric inhibitor, so sucrose synthesis is tightly linked to the G6P/Pi ratio. G6P also increases the activity of SPS via regulation of the PKIII protein kinase (Huber & Huber 1996; Toroser *et al*. 2000). SnRK1, which activates sucrose synthase, is inhibited by G6P (Smeekens *et al*. 2010). SnRK1 is also necessary for sucrose‐dependent redox activation of ADP‐glucose pyrophosphorylase and is therefore a significant regulator of starch accumulation (Kanegae *et al*. 2005; Jain *et al*. 2008). G6P inhibition of SnRK1 could therefore have a significant effect on metabolism as it provides a convergence point for regulatory networks (Cho *et al*. 2010). By altering the relative concentrations of G6P (and hence other metabolites), GPT2 may thus affect a number of metabolic signals controlling gene expression and/or protein synthesis.

There are significant differences between the two lines in terms of other metabolites that respond to HL treatment. T6P has previously been implicated in the regulation of photosynthesis via a feedback loop involving sucrose and SnRK1, which acts to activate expression of photosynthetic genes. T6P is increased by high sucrose levels (Lunn *et al*. 2006) and has been shown to inhibit SnRK1 *in vitro* (Zhang *et al*. 2009; Delatte *et al*. 2011; Nunes *et al*. 2013); however, our data show that there is no simple causal relationship between T6P and photosynthetic capacity. HL induces a much larger transient increase in T6P in *gpt2.2* than in wild‐type plants, yet no acclimation of *P*_max_ occurs in the former case. On the contrary, final steady‐state concentrations of T6P do correlate with photosynthetic capacity. In the mutant, T6P levels return to control levels (Fig. 4) and *P*_max_ is largely unaltered (Athanasiou *et al*. 2010). The wild‐type plant maintains a higher T6P concentration at the end of acclimation and has an *increased* photosynthetic rate and capacity. There is also evidence that T6P is involved in the regulation of starch turnover in the leaf, with increased T6P during the day leading to small increases in starch accumulation and substantial inhibition of starch degradation at night (Martins *et al*. 2013). However, other factors play important roles in the regulation of starch content and turnover (Pyl *et al*. 2012; Martins *et al*. 2013), so it is difficult to interpret these changes in T6P and starch content in isolation. It has recently been proposed that the ratio of T6P:sucrose provides a homeostatic signal allowing plants to maintain appropriate cellular sucrose (Yadav *et al*. 2014). There is a clear correlation over the first 3 d of HL between sucrose and T6P levels, with both increasing in parallel (Figs 2, 4); however, this correlation breaks down on day 5, when sucrose continues to increase while T6P decreases. This might indicate adjustment of the set point for the sucrose:T6P ratio as the plant acclimates to HL. Although GPT2 expression is important for acclimation of photosynthetic capacity to increased light, it is clearly not required for all components of HL acclimation. There is a clear acclimation of metabolism in both the Ws‐4 and Col‐0 wild‐type and the *gpt2* mutants, although global analysis by transcriptomics, FTIR spectroscopy and GC‐MS shows that the nature of this acclimation is different in different cases. The identified intermediates in carbon metabolism show a qualitatively similar response, with steady‐state concentrations of these typically, although not universally, rising initially and then falling to low levels as the plants acclimate to HL. Starch synthesis shows a significant increase in both wild‐type and *gpt2.2* plants, although the wild type retains a greater capacity for starch accumulation even after 7‐day‐acclimation period (see Supporting Information Fig. S4). In contrast, the diurnal accumulation of sucrose remained constant throughout HL treatment.

Within primary metabolism, there is a striking dichotomy of response to HL, with metabolites involved in photosynthesis and glycolysis increasing on the first day at HL, while those involved in the TCA cycle decrease (Fig. 5). These data suggest that the conversion of PEP to pyruvate, catalysed by pyruvate kinase, is a key regulatory step that directly responds to HL (Paul & Pellny 2003). Conversion of PEP to shikimate in the chloroplast may also be important in controlling fluxes through the glycolysis pathway. Shikimate concentrations increased in response to HL, while it is constitutively high in the *gpt2.2* mutant. The return of most metabolite pools to near LL levels following acclimation is consistent with metabolic acclimation operating to achieve an overall homeostasis of metabolism. This occurs in both wild‐type and *gpt2.2* plants, although the rates of change and absolute levels of metabolites are different.

In conclusion, GPT2 allows plants to control the diversion of carbon into transient starch reserves and to regulate the concentrations of various metabolites, notably G6P. These metabolites may not act directly as signals for acclimation; their concentrations change less in acclimating than non‐acclimating plants. Rather, we suggest that the control of metabolite concentrations may provide permissive conditions, allowing photosynthetic acclimation. Expression of GPT2 acts to buffer metabolic changes during acclimation to HL and therefore provides an essential route for antero‐ and retrograde signalling between chloroplast and cytosol.

## Supporting information


**Figure S1.** Photosynthetic rates of plants of Col‐0 (circles) and *gpt2.1* (triangles) measured under growth conditions.
**Figure S2.** MapMan representation of genes showing significant changes in expression in *gpt2.2* leaves, compared to Ws‐4 wild type in low light.
**Figure S3.** MapMan representation of genes showing significant changes in expression in Ws‐4 wild type following transfer from low to high light.
**Figure S4.** MapMan representation of genes showing significant changes in expression in *gpt2.2* following transfer from low to high light.
**Figure S5.** FT‐IR and GC‐MS analysis of changes in metabolism during acclimation to high light.

**Table S1.** Microarray analyses were carried out as in Athanasiou *et al*. (2010).

**Table S2.** Metabolite levels were obtained using GC‐EI‐TOF/MS on tissue from plants under acclimation conditions.

**Table S3.** Metabolite levels were obtained using LC‐MS/MS on tissue from plants under acclimation conditions.
