# Supplementary material for: Acclimation of metabolism to light in A rabidopsis thaliana: the glucose 6‐phosphate/phosphate translocator GPT2 directs metabolic acclimation
Source: Plant Cell Environ. 2015 Jan 25;38(7):1404–17. doi: 10.1111/pce.12495 (PMC4949648; doi:10.1111/pce.12495)
Supplement: Supplementary file 1 — Figure S1. Photosynthetic rates of plants of Col‐0 (circles) and gpt2.1 (triangles) measured under growth conditions. Figure S2. MapMan representation of genes showing significant changes in expression in gpt2.2 leaves, compared to Ws‐4 wild type in low light. Figure S3. MapMan representation of genes showing significant changes in expression in Ws‐4 wild type following transfer from low to high light. Figure S4. MapMan representation of genes showing significant changes in expression in gpt2.2 following transfer from low to high light. Figure S5. FT‐IR and GC‐MS analysis of changes in metabolism during acclimation to high light. [file PCE-38-1404-s001.docx]

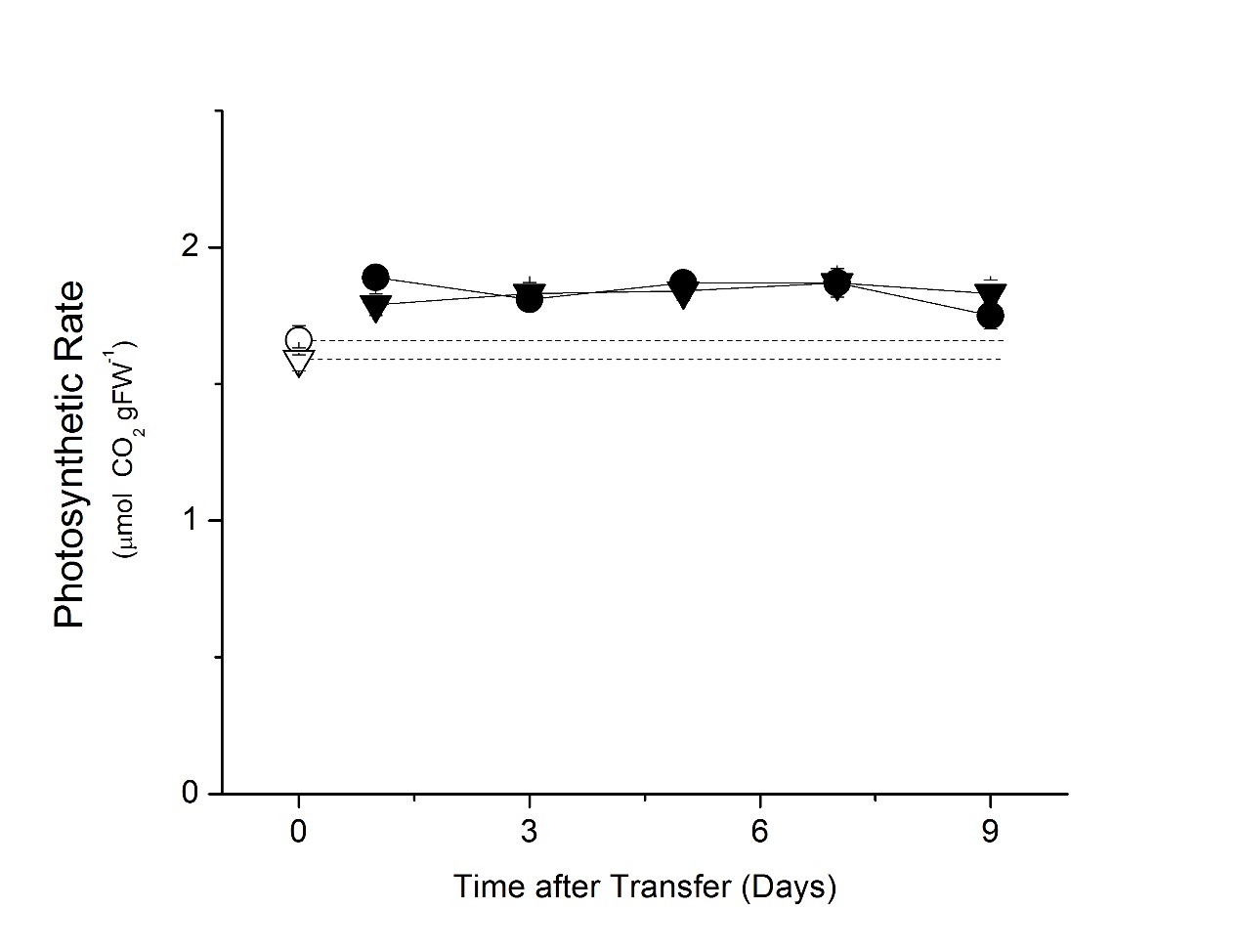


**Supporting Information Figure S1: Photosynthetic rates of plants of Col-0 (circles) and *gpt2.1* (triangles) measured under growth conditions.** Plants were grown at 100 µmol m^-2^ s^-1^ for 8 weeks and the photosynthetic rate measured at the end of day (open symbols). Plants were then transferred to 400 µmol m^-2^ s^-1^ and measured at the end of the photoperiod for up to 9 days (closed symbols). Each point represents the mean +/- s.e. of at least 5 replicates. Where error bars are not visible, they are smaller than symbols.


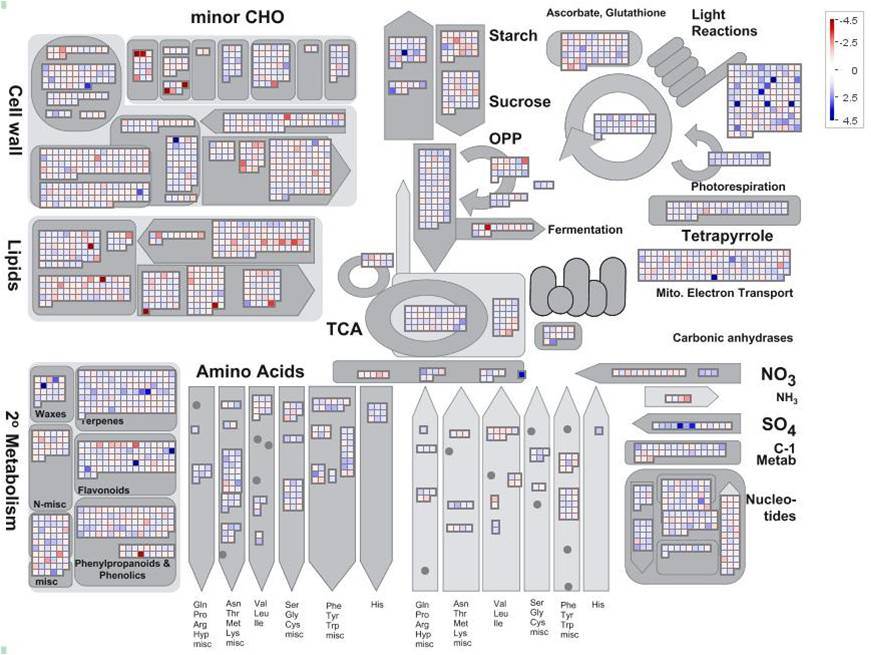


**Supporting information Figure S2: Mapman representation of genes showing significant changes in expression in *gpt2.2* leaves, compared to Ws-4 wild type in low light.** Fully expanded leaves were collected at the mid-point of an 8 hour photoperiod from plants grown for 8 weeks at 100 µmol m^-2^ s^-1^ Gene expression was considered to be significantly different if signal varied by greater than 1.2 fold and P<0.01. For full data set, see Supporting Information Table S1


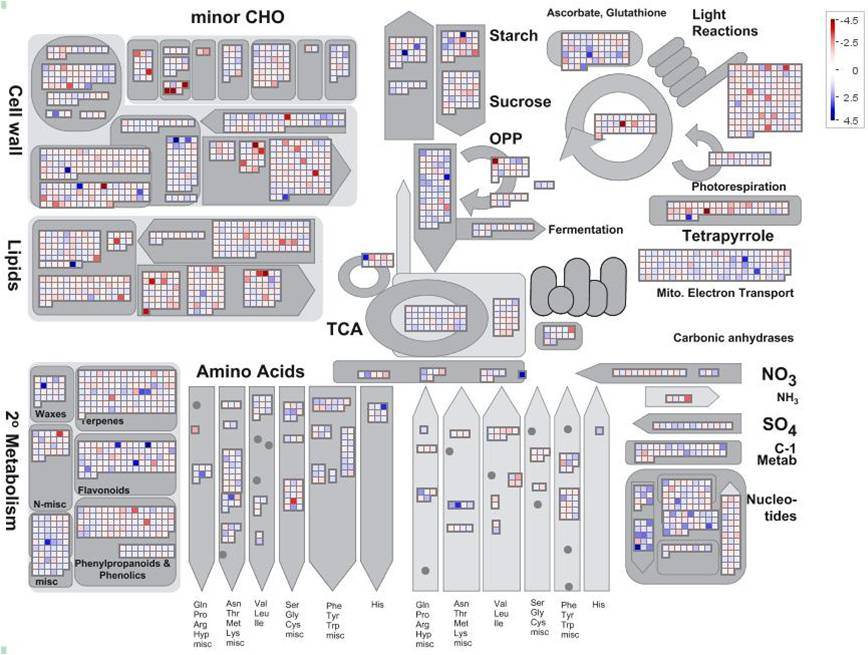


**Supporting information Figure S3: Mapman representation of genes showing significant changes in expression in Ws-4 wild type following transfer from low to high light.** Fully expanded leaves were collected at the mid-point of an 8 hour photoperiod from plants grown for 8 weeks at 100 µmol m^-2^ s^-1^ or after 4 hours exposure to 400 µmol m^-2^ s^-1^ light. Gene expression was considered to be significantly different if signal varied by greater than 1.2 fold and P<0.01. For full data set, see Supporting Information Table S1


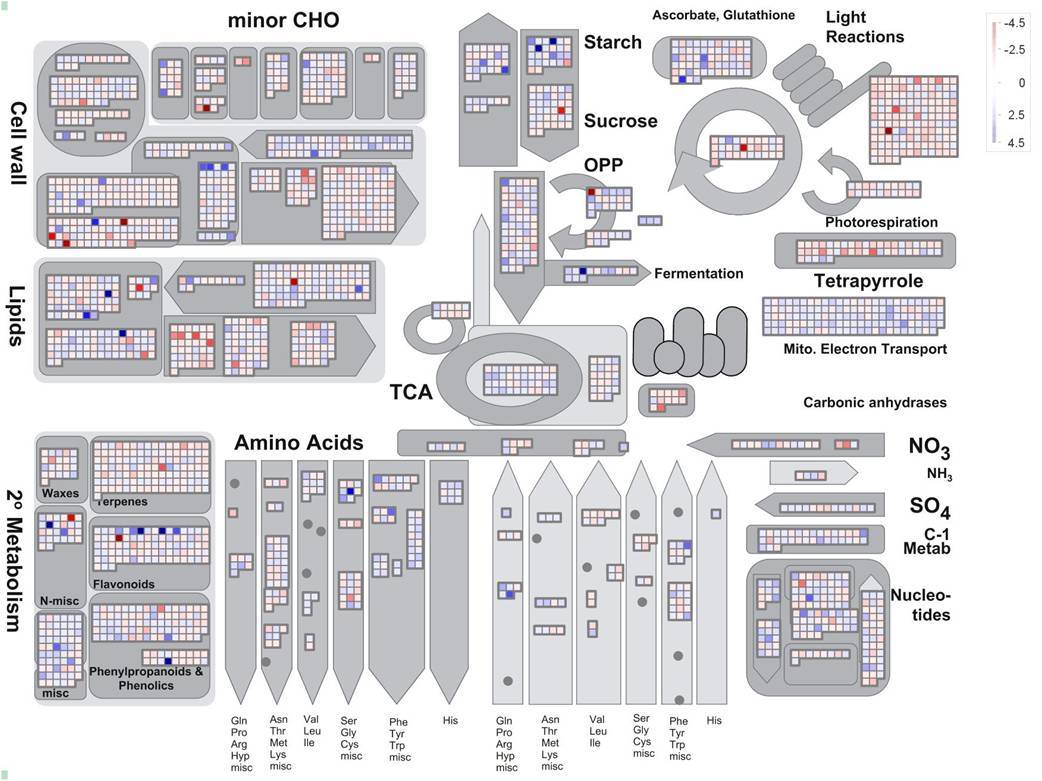


**Supporting information Figure S4: Mapman representation of genes showing significant changes in expression in *gpt2.2* following transfer from low to high light.** Fully expanded leaves were collected at the mid-point of an 8 hour photoperiod from plants grown for 8 weeks at 100 µmol m^-2^ s^-1^ or after 4 hours exposure to 400 µmol m^-2^ s^-1^ light. Gene expression was considered to be significantly different if signal varied by greater than 1.2 fold and P<0.01. For full data set, see Supporting Information Table S1


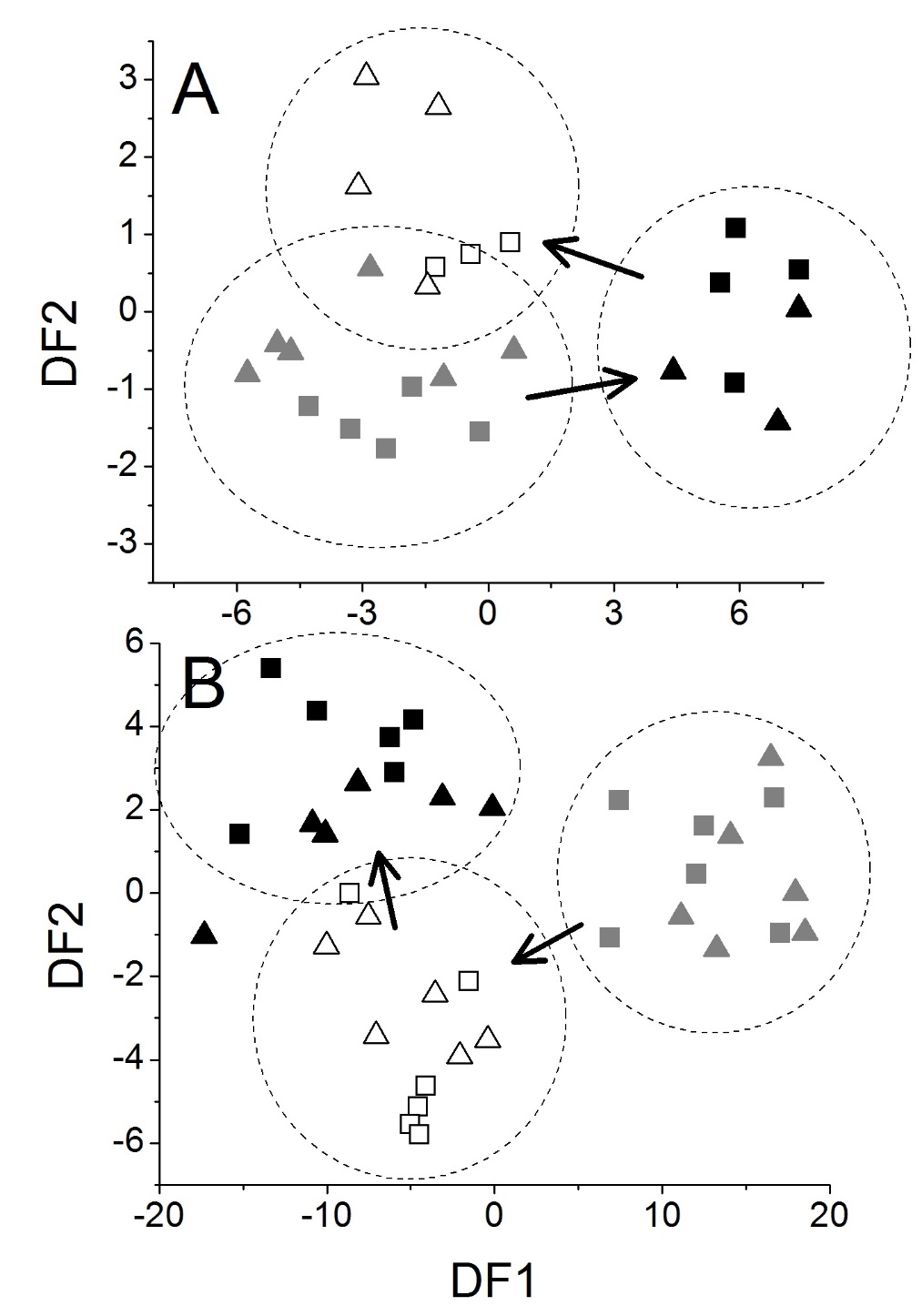


**Figure S5**. **FT-IR and GC-MS analysis of changes in metabolism during acclimation to high light.** (A) PC-DFA model of FT-IR spectra of extracts from Col-0 wild-type and *gpt2.1* lines in the first three days after transfer to HL conditions. Circles and arrows are indicative of clustering only and have no statistical significance. Plants were grown for 8 weeks at low light (100 μmol m ^-2^ s ^-1^) ^­^and then transferred to high light (400 µmol m^-2^ s^-1^) for up to 3 days. 10 PCs were used in the calculation, accounting for 98.5% of the total variance. PC-DF1 explained 47.4% of the total variance, with PC-DF2 explaining a further 22% of the total variance. Grey symbols represent plants at the end of day in LL, black symbols represent plants at the end of 1 photoperiod of HL. Open symbols represent plants at the end of 3 days of HL treatment. (B) PC-DFA model constructed from the GC-MS data from extracts from Col-0 wild-type and *gpt2.1* lines in the first three days after transfer to HL conditions. Samples were collected at the end of the photoperiod in plants maintained at low light (grey symbols) and transferred to HL for 1 (black symbols) and 3 (open symbols) days.
